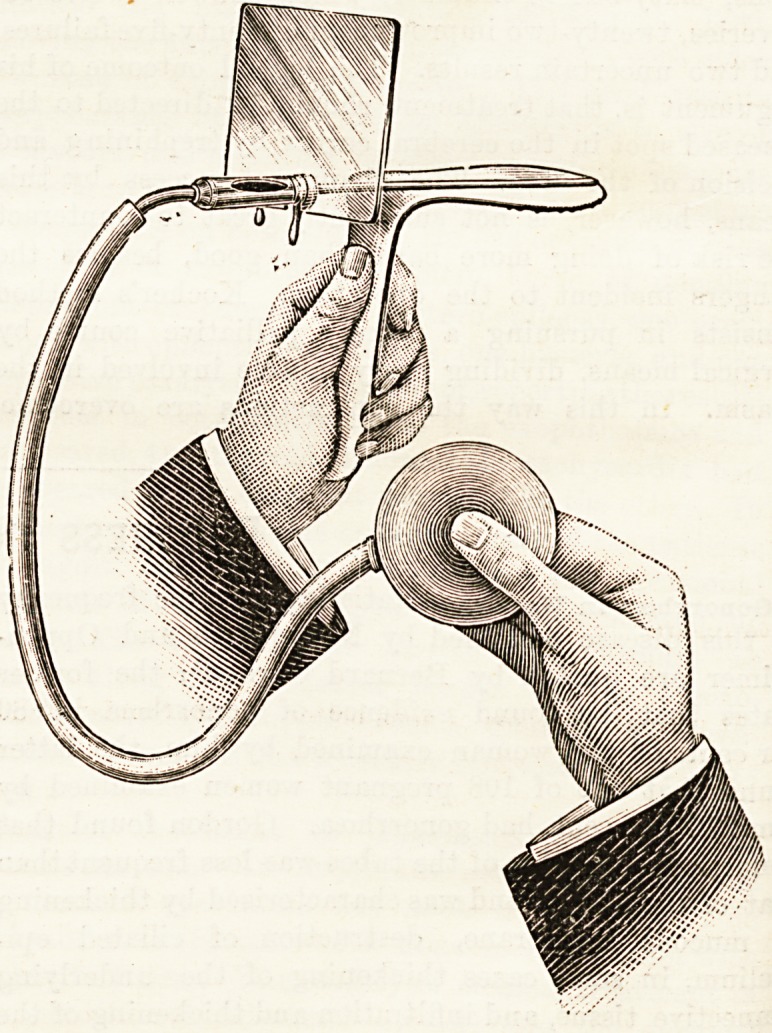# New Appliances and Things Medical

**Published:** 1897-04-17

**Authors:** 


### NEW APPLIANCES AND THINGS MEDICAL.

### HALL'S COCA WINE.

(Stephen Smith and Co., Bow.)

Owing to the recent discussion on the merits and demerits of Coca Wines, we have had submitted to us further samples of this particular brand, and have made a special examination of it with a view of ascertaining whether this well-known wine deserves its high reputation for uniform and genuine quality. The samples possessed a powerful herbaceous aroma, and the vinous and fruity taste which is so characteristic of coca wine. An examination of the colouring matter satisfied our analyst that it was a genuine wine-colouring matter, but he also detected a small amount of chlorophyll, derived no doubt from the colouring matter of the coca leaves used in the preparation. The quantity of alcohol was 13·00 per cent. absolute alcohol by weight, corresponding to 28·13 per cent. proof spirit. This quantity of alcohol is consistent with a genuine unfortified wine. The total acidity calculated to tartaric acid amounted to 0·63 per cent., and of this amount 0·487 per cent. was fixed. The total extract was 10·43 per cent., and the wine left 0·39 per cent. of mineral ash on ignition. A careful determination of the amount of alkaloids present showed that the quantity present could have been derived by macerating 4·4 grams of average coca leaves in 100 c.c. of the wine. This quantity is slightly less than that stated by the proprietors, if one takes a wine glass to be equivalent to two fluid ounces. From our examination we are of the opinion that Hall's Coca Wine is what it purports to be, viz., a genuine wine, in which the active principles of the erythroxylon coca have been incorporated by maceration of the leaves of this well-known drug in a good, sound, genuine red wine.

### COMBINED TONGUE DEPRESSOR AND INSUFFLATOR.

(James Woolley, Sons, and Co., Limited, Manchester.)

This handy little instrument is the invention of Dr. W. Thomas, of Bridgend, Glamorganshire A tube in connection with the india rubber tubing and ball runs along the length of the horizontal limb of the tongue depressor. At the proximal end of the metal tube is a chamber in which can be placed the powder to be used for insufflation. The instrument can be provided with a glass plate shield for the protection of the operator in dealing with diphtheritic and other infectious cases. The instrument is made throughout of nickel plated metal, and can be conveniently cleaned and rendered aseptic. The only objection that we can see to this handy little insufflator is that the insufflated powder has not sufficient "spread," in fact in certain cases it is somewhat difficult to direct the application on a particular area of the pharynx. This, however, is a minor point, and compared with other forms of insufflator this instrument does its work far more satisfactorily.

## Figures and Tables

**Figure f1:**